# Tumor apparent diffusion coefficient as a predictive marker for PD-1 inhibitor outcome in advanced cervical cancer: a retrospective study

**DOI:** 10.3389/fonc.2026.1769071

**Published:** 2026-03-27

**Authors:** Yanjie Yang, Guilin Zhang, Haixiao Shang, Xiaona Fu, Jie Lou, Shanshan Jiang, Weiwei Liu, Yi Li, Yuan Zhang, Lian Yang

**Affiliations:** 1 Department of Radiology, Union Hospital, Tongji Medical College, Huazhong University of Science and Technology, Wuhan, China; 2 Hubei Provincial Clinical Research Center for Precision Radiology & Interventional Medicine, Wuhan, China; 3 Hubei Key Laboratory of Molecular Imaging, Wuhan, China; 4 Department of Obstetrics and Gynecology, Union Hospital, Tongji Medical College, Huazhong University of Science and Technology, Wuhan, China

**Keywords:** apparent diffusion coefficient, diffusion-weighted imaging, immunotherapy, predictive biomarker, uterine cervical neoplasms

## Abstract

**Background:**

To investigate whether pre-treatment apparent diffusion coefficient (ADC) values from diffusion-weighted imaging (DWI) predict clinical outcomes in patients with advanced or locally advanced cervical cancer undergoing Programmed Death-1 (PD-1) inhibitor therapy. Despite the growing use of immunotherapy, reliable and non-invasive imaging biomarkers to predict treatment response in cervical cancer are lacking.

**Methods:**

This retrospective study analyzed 167 cervical cancer patients treated with PD-1 inhibitors at Wuhan Union Hospital between 01/05/2020 and 31/12/2023. The whole tumor ADC (wADC) and substantial tumor ADC (sADC; excluding necrotic, cystic, and vascular regions) were measured on the largest tumor slice. Optimal cut-off values for ADC and inflammatory markers were determined using X-tile software based on overall survival (OS). Progression-free survival (PFS) and OS were analyzed using Kaplan-Meier curves and Cox regression models.

**Results:**

Lower sADC was associated with significantly worse PFS (hazard ratio [HR] = 1.81; 95% confidence interval [CI]: 1.18-2.80; *P* = 0.005) and OS (HR = 2.40; 95% CI: 1.36-4.21; *P* = 0.002), whereas wADC showed no significant prognostic correlation with PFS (HR = 0.64; 95% CI: 0.39-1.06; *P* = 0.123) and OS (HR = 0.62; 95% CI: 0.32-1.19; *P* = 0.209). Multivariate Cox regression revealed low-sADC as an independent risk factor for OS (HR = 2.15, 95% CI: 1.14-4.03, *P* = 0.017) and PFS (HR = 1.70, 95% CI: 1.08-2.67, *P* = 0.021).

**Conclusion:**

The substantial tumor ADC (excluding necrotic, cystic, and vascular regions) was associated with prognosis in cervical cancer patients receiving PD-1 inhibitor therapy.

## Introduction

Cervical cancer is the fourth most frequently diagnosed cancer and the fourth leading cause of cancer death in women globally, with over 604,000 new cases and 342,000 deaths reported in 2020 (1). Presently, the primary treatment approaches for cervical cancer, including radiotherapy, chemotherapy, and/or surgical removal, are associated with significant side effects and limited efficacy, particularly in advanced-stage disease (2). Recent advances in immunotherapy, particularly immune checkpoint inhibitors, have improved survival outcomes for advanced cervical cancer (3–5). For example, the KEYNOTE-826 randomized clinical trial demonstrated that pembrolizumab combined with chemotherapy (with or without bevacizumab) significantly improved both OS and PFS in patients with persistent, recurrent, or metastatic cervical cancer (4). Although Programmed Death-1 (PD-1) inhibitors are being used more frequently, patient responses to treatment vary considerably. This highlights the need for reliable biomarkers to predict outcomes and guide personalized therapy.

Currently, there is limited research investigating biomarkers that can predict clinical outcomes for cervical cancer patients receiving immune checkpoint inhibitor therapy. In a retrospective study of 102 advanced cervical cancer patients receiving PD-1 inhibitors, Cheng et al. identified squamous cell carcinoma histology, recurrence-free intervals exceeding 6 months, and combination therapy with PD-1 inhibitors as positive prognostic factors (6). Additionally, genomic analyses by Huang et al. revealed that ERBB3 mutations, PD-L1 positivity, and high tumor mutational burden (TMB) were significantly associated with improved survival outcomes in patients treated with PD-1 inhibitor combination therapy (7). The expression levels of programmed death-ligand 1 (PD-L1) have a demonstrated link to the response to immune checkpoint inhibitors (8). However, conventional biomarkers like PD-L1 expression and TMB typically require invasive tissue sampling for assessment and incur high costs. This poses challenges including procedural risks, potential sampling bias due to tumor heterogeneity, limited feasibility for serial monitoring, and variability in testing methodologies. Despite these challenges, significant heterogeneity in treatment responses persists, underscoring the need for further exploration of novel prognostic markers to optimize individualized therapeutic strategies. Currently, there is a critical lack of reliable, non-invasive imaging biomarkers derived from routine clinical MRI to predict the efficacy of PD-1 inhibitor therapy in cervical cancer.

As a widely used functional imaging modality, diffusion-weighted magnetic resonance imaging (DWI) quantifies the mobility of water molecules within tissue to provide insights into the tumors’ cellular microenvironment (9–11). The apparent diffusion coefficient (ADC), derived from DWI, serves as a quantitative biomarker for assessing tumor cellularity and post-therapeutic necrosis (12). Previous studies have demonstrated the utility of ADC values in predicting malignancy and clinical outcomes across cancers (10, 13, 14), including cervical cancer (15–18). For instance, Liu et al. correlated ADC values with PD-L1 expression and proposed a nomogram integrating ADC values and clinicopathological parameters to predict PD-L1 expression in cervical cancer patients (19). While these findings suggest a potential link between ADC and the immune microenvironment, no study has directly investigated whether pre-treatment ADC values can predict clinical outcomes, such as progression-free survival (PFS) and overall survival (OS), in cervical cancer patients receiving PD-1 inhibitor therapy.

This study uniquely investigates ADC values before immunotherapy in patients with locally advanced or advanced cervical cancer, systematically evaluating their predictive power for clinical outcomes. Unlike prior ADC studies focused on chemoradiation, this is the first to link ADC to PD-1 inhibitor outcomes.

## Methods

### Study design and patient selection

We retrospectively enrolled patients with locally advanced or advanced cervical cancer who received PD-1 inhibitor treatment at Wuhan Union Hospital between 01/05/2020 and 31/12/2023. The treatment regimen consisted of a PD-1 inhibitor (200 mg of pembrolizumab, tislelizumab, camrelizumab, or sintilimab) administered every 3 weeks. The inclusion criteria were as follows: (1) histologically confirmed cervical cancer (including squamous cell carcinoma, adenocarcinoma, and other pathological types) based on biopsy; (2) age ≥ 18 years; (3) treatment with PD-1 inhibitor as monotherapy or in combination with chemotherapy, radiotherapy, or anti-angiogenic agents; (4) underwent a pelvic magnetic resonance scan within 7 days before initiating immunotherapy. Patients were excluded based on the following criteria: (1) patients who did not receive the pelvic MRI before the first immunotherapy; (2) patients with other primary malignant tumors; (3) the clinical or prognostic data of patients are incomplete.

Clinical data, including demographic characteristics, tumor-specific details, and laboratory results, were collected retrospectively from electronic medical records. The data were accessed for research purposes between 01/07/2024 and 31/03/2025. The inflammatory indicators included neutrophil-to-lymphocyte ratio (NLR), platelet-to-lymphocyte ratio (PLR), monocyte-to-lymphocyte ratio (MLR), and systemic immune-inflammation index (SII).

This retrospective study adhered to the principles of the Declaration of Helsinki and received ethical approval from the Ethics Committee of Wuhan Union Hospital. Due to the retrospective nature of this study, the ethics committee waived the need for written informed consent. All analyses were conducted using de-identified datasets to protect participant privacy.

### ADC measurement

**Figure 1:**
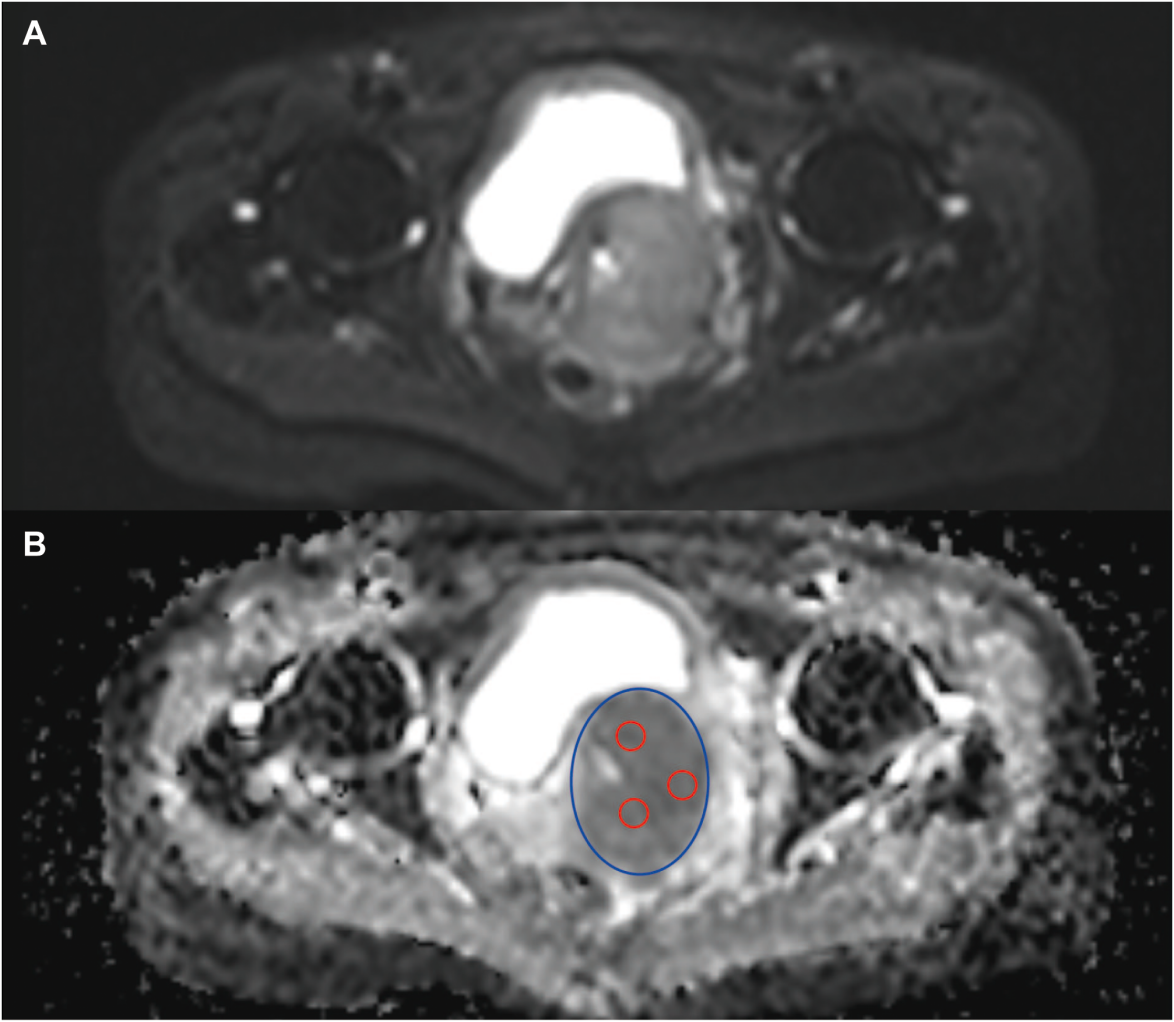
Illustration of the ROI delineation. **(A)** The diffusion-weighted images of the tumor; **(B)** The ROIs of wADC (blue) and sADC (red) on the ADC map. ROI, region of interest; wADC, whole tumor apparent diffusion coefficient; sADC, substantial tumor apparent diffusion coefficient.

All images were independently analyzed by two radiologists (with 5 and 10 years of gynecologic MRI experience, respectively) using the Picture Archiving and Communication System (PACS). The ADC maps were generated automatically by the MRI system and the ADC value was calculated by the following formula: $ADC=\frac{\ln(SI1/SI2)}{\left(b2-b1\right)}$, where SI1 and SI2 indicate the signal intensities acquired at b = b1 and b = b2 s/mm^2^, respectively. The tumor location was determined by contrast-enhanced MRI. The regions of interest (ROIs) were manually outlined on the maximum layer of the tumor, encompassing the entire tumor area (including solid components, cysts, necrosis, and blood vessels). These ROIs were defined as the whole tumor ADC (wADC). Additionally, three ROIs of approximately 100 mm^2^ were manually delineated on the same layer of the tumor, excluding cysts, necrotic areas, and blood vessels, with the average value defined as the substantial tumor ADC (sADC). The selection of three 100 mm^2^ ROIs was designed to ensure adequate sampling of the substantial tumor component. By using standardized 100 mm^2^ circular regions, we balanced the need for capturing representative tissue against the risk of including peripheral partial-volume effects or microscopic necrotic foci that could skew the ADC values. The combined area of these three ROIs represented a substantial and representative portion of the solid tumor area on the largest slice. The measurement protocol is illustrated in **Figure 1**. The final ADC values were determined by averaging the measurements from the two radiologists. Any discrepancies were resolved through consensus.

### Determination of the optimal cut-off value

In this study, we determined the optimal cut-off values for wADC, sADC, and inflammatory indicators using X-tile software (version 3.6.1; Yale University School of Medicine, New Haven, CT, USA). X-tile software is a standard bio-informatics tool for biomarker assessment and outcome-based cut-point optimization, which systematically evaluates all possible cut-off points to segregate the cohort into high- and low-score groups based on their association with OS and PFS (20). The statistical significance of the optimal cut-off was assessed using the log-rank test, with P values adjusted for multiple testing via Monte Carlo simulation to ensure robustness. Based on the above method, the optimal cut-off values determined based on OS are as follows: wADC (cut-off = 1938×10^−6^ mm^2^/s), sADC (cut-off = 1564×10^−6^ mm^2^/s), NLR (cut-off = 4.1), PLR (cut-off = 271.1), MLR (cut-off = 0.6), SII (cut-off = 820.8) (**Supplementary Figure 1**).

### Definition and evaluation of data

All patients were followed up until July 31, 2024. Tumor response was evaluated 6–8 weeks post-treatment using the immune-related Response Evaluation Criteria in Solid Tumors, with outcomes categorized as complete response (CR), partial response (PR), stable disease (SD), or progressive disease (PD) (21). If imaging findings suggested PD, a repeat tumor assessment was performed at least 4 weeks later. Patients who achieved CR or PR were classified as “responders”, while those with PD or SD were labeled as “non-responders”. The objective response rate was defined as the proportion of patients achieving CR or PR, while the disease control rate included patients with CR, PR, or SD. PFS was defined as the time interval from initiation of immunotherapy to tumor progression or death from any cause. OS was measured from the first administration of PD-1 inhibitor therapy until the last follow-up date or death from any cause.

### Statistical analysis

All statistical analyses were performed using R software (version 4.4.3; R Foundation for Statistical Computing), SPSS software (Version 27.0; IBM Corp., Armonk, NY, USA) and MSTATA software (www.mstata.com). All statistical tests were two-tailed, with *P* < 0.05 considered statistically significant. The Wilcoxon rank-sum test, independent t-test, or the χ2 test was used to compare the baseline characteristics between different groups. Inter-observer agreement for ADC measurements was evaluated by the ICC, with ICC > 0.75 indicating good agreement.

PFS and OS differences between high/low-wADC and high/low-sADC groups were analyzed with Kaplan-Meier curves and the log-rank test. Variables with *P* < 0.1 in univariate analysis were entered into the multivariate Cox proportional hazards model. The proportional hazards assumption was verified using the cox.zph function (R survival package), with all variables meeting the assumption (*P* > 0.05).

Model performance was evaluated using the concordance index (C-index). Subgroup hazard ratios were estimated via unstratified Cox regression and visualized in forest plots. Given the exploratory design, no multiplicity adjustment was applied.

## Results

### Clinical characteristics

We retrospectively collected a total of 224 cervical cancer cases treated with PD-1 inhibitors at our center from May 2020 to December 2023. Among these cases, 21 patients lacked available prognostic data, and an additional 36 patients did not undergo pelvic MRI before initiating immunotherapy. Finally, 167 cervical cancer patients were included in this study. ICC analysis demonstrated good inter-observer agreement for both wADC (ICC = 0.878; *P* < 0.001) and sADC (ICC = 0.873; *P* < 0.001) measurements. In addition, the variability among the three localized ROIs used for sADC measurement was evaluated. The mean coefficient of variation (CV) for the three ROI measurements across all patients was 12.3%, indicating high consistency in the sampling of solid tumor regions.

Using X-tile software, all patients were stratified into high-wADC (n = 32) and low-wADC (n = 135) groups based on wADC values, and into high-sADC (n = 91) and low-sADC (n = 76) groups based on sADC values. Among 167 cervical cancer patients, 22 (13.2%) patients received PD-1 inhibitors plus chemotherapy, 101 (60.5%) patients received PD-1 inhibitors with chemoradiotherapy, and 44 (26.3%) received PD-1 inhibitors combined with chemotherapy and antiangiogenic therapy.

The clinical characteristics of patients in the high-sADC and low-sADC groups are summarized in **Table 1**. Overall, the characteristics of the patients including demographics, tumor-related information, and biochemical parameters were similar between the two groups.

**Table 1:** The clinical characteristics of patients in the high-sADC and low-sADC groups.

Characteristics	High-sADC (n = 91)	Low-sADC (n = 76)	*P* value
Age, n (%)			0.294
< 50 years	21 (12.6%)	23 (13.8%)	
≥ 50 years	70 (41.9%)	53 (31.7%)	
BMI, n (%)			0.913
≥ 25 kg/m^2^	21 (12.6%)	17 (10.2%)	
< 25 kg/m^2^	70 (41.9%)	59 (35.3%)	
FIGO stage, n (%)			0.670
II	23 (13.8%)	15 (9.0%)	
III	34 (20.4%)	32 (19.2%)	
IV	34 (20.4%)	29 (17.4%)	
Pathological type, n (%)			0.800
Squamous cell carcinoma	69 (41.3%)	55 (32.9%)	
Adenocarcinoma	16 (9.6%)	14 (8.4%)	
Others	6 (3.6%)	7 (4.2%)	
Diabetes, n (%)			0.918
No	86 (51.5%)	73 (43.7%)	
Yes	5 (3.0%)	3 (1.8%)	
Hypertension, n (%)			0.249
No	83 (49.7%)	65 (38.9%)	
Yes	8 (4.8%)	11 (6.6%)	
TG (mmol/L), median (IQR)	1.4 (1.1, 1.8)	1.3 (0.9, 1.9)	0.448
TB (mg/dL), median (IQR)	9.2 (7.1, 12.4)	8.5 (7.1, 10.0)	0.048
Albumin (g/L), mean ± SD	39.9 ± 3.6	39.1 ± 4.0	0.181
NLR, n (%)			0.142
< 4.1	66 (39.5%)	47 (28.1%)	
≥ 4.1	25 (15.0%)	29 (17.4%)	
PLR, n (%)			0.982
< 271.1	66 (39.5%)	55 (32.9%)	
≥ 271.1	25 (15%)	21 (12.6%)	
MLR, n (%)			0.395
< 0.6	76 (45.5%)	67 (40.1%)	
≥ 0.6	15 (9.0%)	9 (5.4%)	
SII, n (%)			0.021
< 820.8	68 (40.7%)	44 (26.3%)	
≥ 820.8	23 (13.8%)	32 (19.2%)	

sADC, substantial tumor apparent diffusion coefficient; BMI, body mass index; FIGO, International Federation of Gynecology and Obstetrics; TG, triglyceride; TB, total bilirubin; IQR, interquartile range; NLR, neutrophil to lymphocyte ratio; PLR, platelet to lymphocyte ratio; MLR, monocyte to lymphocyte ratio; SII, systemic immune-inflammation index.

### Tumor response and survival outcomes

The median follow-up time for all patients was 17.1 months (95% CI: 15.9-19.3). The overall ORR and DCR were 34.1% and 85.0%, respectively. The median PFS was 20.0 months (95% CI: 13.2-30.0), while the median OS was not reached. Tumor responses in the high-sADC and low-sADC groups were similar, as presented in **Supplementary Table 1**. The objective response rate (25.3% vs. 38.2%, *P* = 0.073) and disease control rate (87.9% vs. 81.6%, *P* = 0.253) did not differ significantly between the groups. During the follow-up period, 19 patients (20.9%) in the high-sADC group and 31 patients (40.8%) in the low-sADC group died.

**Figure 2:**
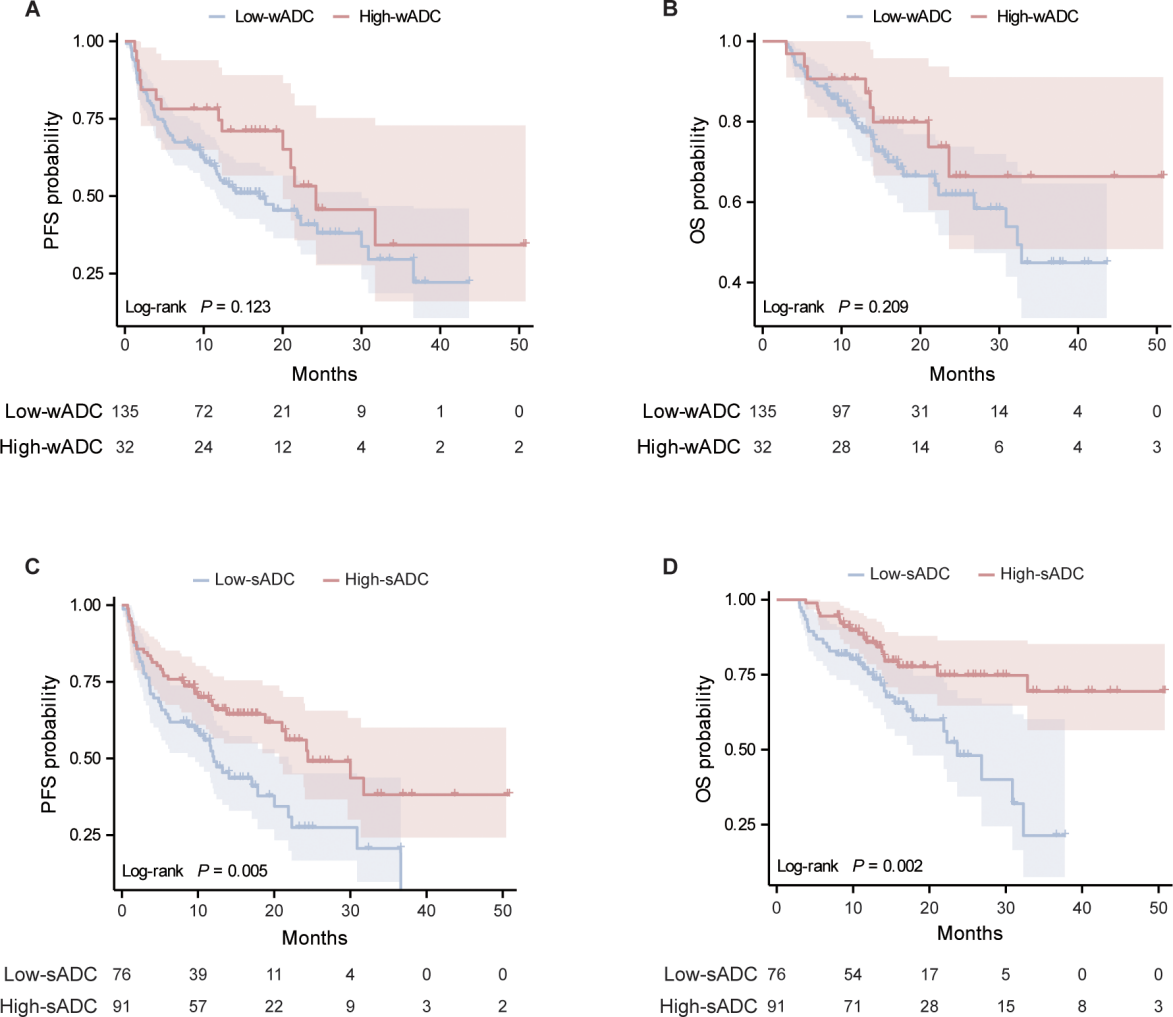
Kaplan-Meier curves of PFS **(A)** and OS **(B)** in the low-wADC group (blue) and high-wADC (red) group; Kaplan-Meier curves of PFS **(C)** and OS **(D)** in the low-sADC group (blue) and high-sADC (red) group. PFS, progression-free survival; OS, overall survival; wADC, whole tumor apparent diffusion coefficient; sADC, substantial tumor apparent diffusion coefficient.

**Figure 3:**
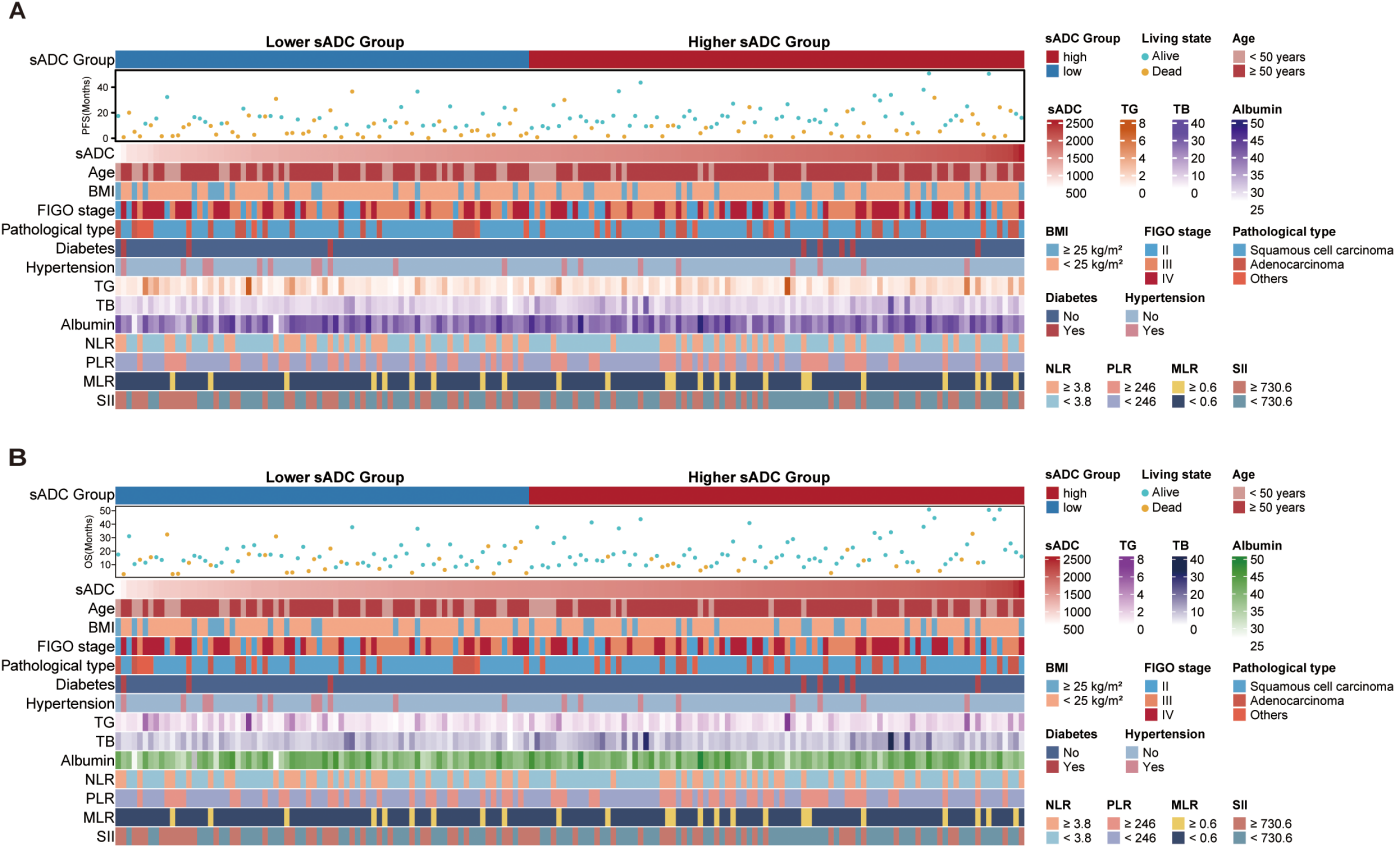
Heatmaps shows the relationship between sADC, clinical features, PFS **(A)** and OS **(B)**. sADC, substantial tumor apparent diffusion coefficient; PFS, progression-free survival; OS, overall survival; BMI, body mass index; FIGO, International Federation of Gynecology and Obstetrics; TG, triglyceride; TB, total bilirubin; NLR, neutrophil-to-lymphocyte ratio; PLR, platelet-to-lymphocyte ratio; MLR, monocyte-to-lymphocyte ratio; SII, systemic immune-inflammation index.

Comparing the high-wADC and low-wADC groups, the Kaplan-Meier curves for PFS and OS were illustrated in **Figures 2A, B**, respectively. The results of the Log-rank test indicated no statistically significant difference in survival curves between the two groups for both PFS (hazard ratio [HR] = 0.64; 95% confidence interval [CI]: 0.39-1.06; *P* = 0.123) and OS (HR = 0.62; 95% CI: 0.32-1.19; *P* = 0.209). Regarding the high-sADC and low-sADC groups, Kaplan-Meier curve analysis, as shown in **Figures 2C, D** revealed that the low-sADC group had significantly worse PFS (HR = 1.81; 95% CI:1.18-2.80; *P* = 0.005) and OS (HR = 2.40; 95% CI: 1.36-4.21; *P* = 0.002) compared to the high-sADC group. In addition, the relationship among sADC, clinical features, and survival outcomes of patients in different sADC groups is shown in heatmaps (**Figure 3**).

### Cox regression analysis and subgroup analysis

To further investigate the prognostic significance of sADC in cervical cancer patients, this study performed both univariate and multivariate COX regression analyses to evaluate its association with survival outcomes. Multivariate COX regression analysis revealed that FIGO stage III (HR = 3.42; *P* < 0.001) and IV (HR = 3.76; *P* < 0.001), other pathological types (HR = 2.38; *P* = 0.017), NLR < 4.1 (HR = 1.80; *P* = 0.009), SII < 820.8 (HR = 2.33; *P* < 0.001), and low-sADC (HR = 1.83; *P* = 0.006) were significantly associated with shorter PFS in cervical cancer patients treated with PD-1 inhibitors (**Supplementary Table 2**).

Furthermore, FIGO stage III (HR = 7.80; *P* = 0.001) and IV (HR = 6.49; *P* = 0.003), other pathological types (HR = 3.61; *P* = 0.004), NLR ≥ 4.1 (HR = 2.23; *P* = 0.042), MLR ≥ 0.6 (HR = 2.37; *P* = 0.033), SII ≥ 820.8 (HR = 2.76; *P* = 0.011), and low-sADC (HR = 2.15; *P* = 0.017) were identified as independent predictors of OS (**Table 2**). The multivariate model for OS demonstrated good discriminatory ability, with a C-index of 0.780 (95% CI: 0.747-0.812).

**Table 2:** Univariate and multivariate cox proportional hazards analyses for OS in cervical cancer patients.

Variables	Univariate analysis		Multivariate analysis	
	Hazard ratio (95% CI)	*P* value	Hazard ratio (95% CI)	*P* value
Age				
< 50 years	Reference			
≥ 50 years	1.23 (0.64 - 2.36)	0.534		
BMI				
< 25 kg/m^2^	Reference			
≥ 25 kg/m^2^	0.68 (0.32 - 1.46)	0.326		
FIGO stage				
II	Reference		Reference	
III	6.09 (1.83 - 20.23)	**0.003**	7.80 (2.29 - 26.56)	**0.001**
IV	5.98 (1.78 - 20.09)	**0.004**	6.49 (1.90 - 22.20)	**0.003**
Pathological type				
Squamous cell carcinoma	Reference		Reference	
Adenocarcinoma	0.75 (0.33 - 1.69)	0.486	1.02 (0.44 - 2.35)	0.963
Others	2.54 (1.12 - 5.77)	**0.026**	3.61 (1.50 - 8.69)	**0.004**
Diabetes				
No	Reference			
Yes	2.28 (0.81 - 6.36)	0.117		
Hypertension				
No	Reference			
Yes	0.79 (0.31 - 1.99)	0.621		
TG	1.04 (0.83 - 1.31)	0.744		
TB	0.94 (0.87 - 1.01)	0.088	0.99 (0.91 - 1.07)	0.749
Albumin	1.01 (0.94 - 1.09)	0.783		
NLR				
< 4.1	Reference		Reference	
≥ 4.1	4.26 (2.38 - 7.63)	**< 0.001**	2.23 (1.03 - 4.81)	**0.042**
PLR				
< 271.1	Reference		Reference	
≥ 271.1	2.10 (1.18 - 3.76)	**0.012**	0.66 (0.32 - 1.37)	0.268
MLR				
< 0.6	Reference		Reference	
≥ 0.6	2.03 (1.01 - 4.09)	**0.046**	2.37 (1.07 - 5.26)	**0.033**
SII				
< 820.8	Reference		Reference	
≥ 820.8	4.20 (2.36 - 7.49)	**< 0.001**	2.76 (1.26 - 6.06)	**0.011**
Treatment pattern				
PD-1 + chemotherapy	Reference			
PD-1 + radiochemotherapy	1.241 (0.482 – 3.193)	0.655		
PD-1 + chemotherapy + anti-angiogenic therapy	1.057 (0.379 – 2.948)	0.915		
sADC Group				
High-sADC	Reference		Reference	
Low-sADC	2.44 (1.37 - 4.36)	**0.002**	2.15 (1.14 - 4.03)	**0.017**

OS, overall survival; HR, hazard ratio; CI, confidence interval; BMI, body mass index; FIGO, International Federation of Gynecology and Obstetrics; TG, triglyceride; TB, total bilirubin; NLR, neutrophil to lymphocyte ratio; PLR, platelet to lymphocyte ratio; MLR, monocyte to lymphocyte ratio; SII, systemic immune-inflammation index; PD-1, programmed death-1; sADC, substantial tumor apparent diffusion coefficient.

Values in bold indicate statistical significance.

**Figure 4:**
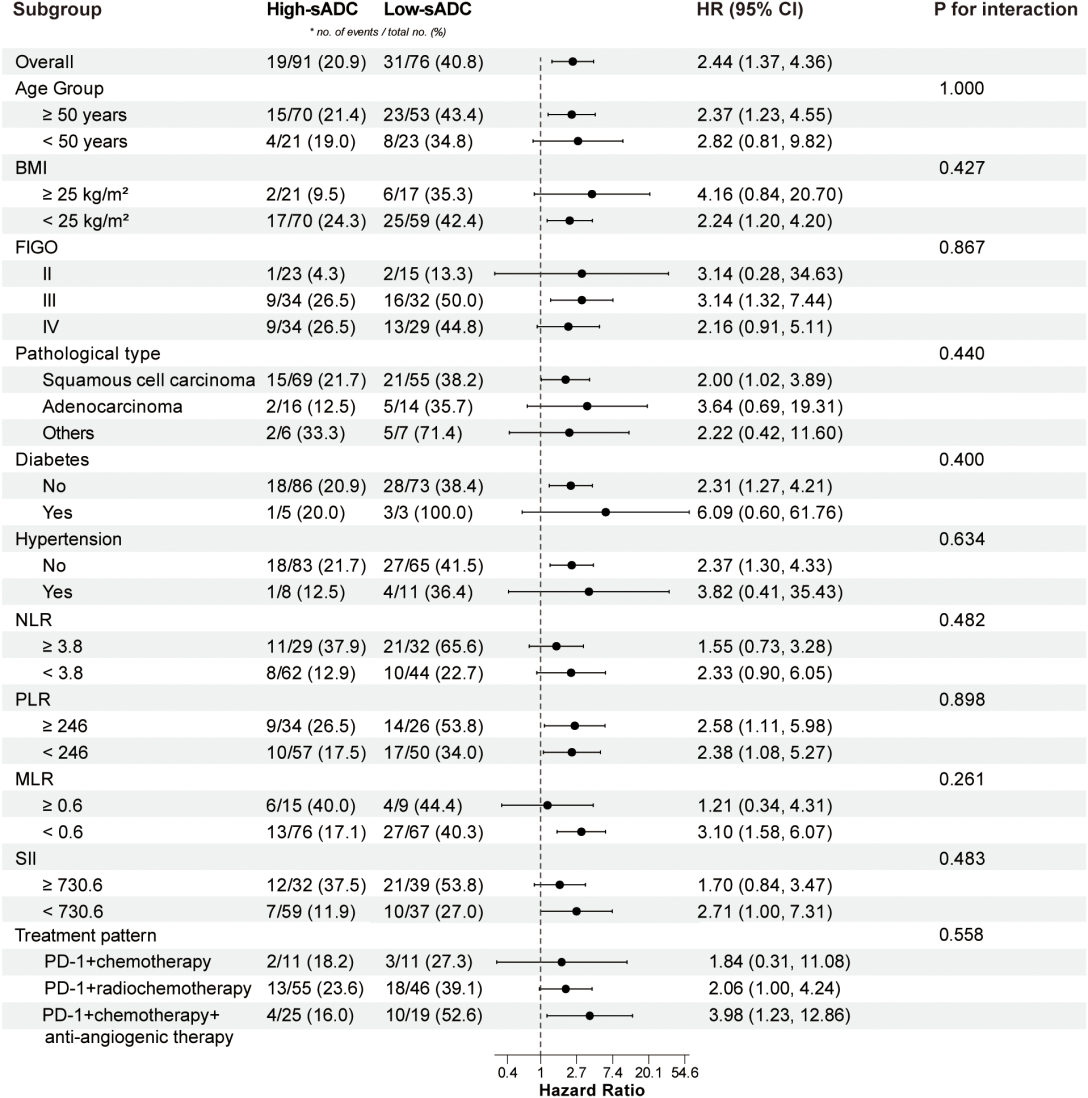
Forest plot of subgroup analysis in OS between the high-sADC group and the low-sADC group. sADC, substantial tumor apparent diffusion coefficient; OS, overall survival; HR, hazard ratio; CI, confidence interval; BMI, body mass index; FIGO, International Federation of Gynecology and Obstetrics; NLR, neutrophil to lymphocyte ratio; PLR, platelet to lymphocyte ratio; MLR, monocyte to lymphocyte ratio; SII, systemic immune-inflammation index; PD-1, programmed death-1.

In the PFS analysis by patient subgroups, low-sADC patients showed worse outcomes than high-sADC patients in most analyzed subgroups, although statistical significance was not reached in some subgroups (**Supplementary Figure 2**). Similarly, **Figure 4** illustrates the association between sADC levels and OS across different patient subgroups. The analysis suggested that lower sADC may be a potential risk factor for OS. Notably, no significant interaction was observed between high and low sADC subgroups in either the OS or PFS subgroup analyses (*P* > 0.05).

## Discussion

This study specifically investigated patients with advanced or locally advanced cervical cancer undergoing PD-1 inhibitor therapy, conducting a comprehensive comparison between two distinct ROI delineation approaches in pre-treatment DWI: the whole tumor versus the substantial tumor analysis. Our results demonstrated that sADC measurements exhibited a strong prognostic correlation with both PFS and OS, whereas wADC values incorporating heterogeneous non-viable tumor components showed no significant predictive value. These findings not only confirm that ROI selection critically impacts ADC quantification reliability (19, 22, 23), but also suggest that sADC better reflects the pathophysiologic tumor microenvironment relevant to immunotherapy response.

Generally, due to the hindrance of cell membranes, water molecule diffusion is restricted in tissues with high cellular density. Previous studies have indicated that malignant tumors often exhibit lower apparent diffusion coefficient (ADC) values (9, 15, 16, 24–26), which is attributed to their characteristic of high cellular density. In contrast, in tumor necrosis areas, the disintegration and destruction of cellular structures lead to an increase in the diffusion of free water molecules, resulting in a significant increase in ADC values (27). Therefore, when the ROIs include necrotic or cystic areas, diffusion of free water molecules within these regions can cause a false elevation of ADC values, preventing them from truly reflecting the density distribution of tumor cells and the state of cell membrane integrity. Moreover, the inclusion of blood vessels may further interfere with measurements due to blood flow artifacts. This study precisely excludes these interfering regions including necrotic areas, cystic areas, and vascular structures, enabling the sADC value to more accurately reflect the microscopic structural features of solid tumor tissues. Multivariate regression analysis further revealed a significant correlation between sADC stratification and immunotherapy response, suggesting that sADC may serve as a novel imaging biomarker for predicting immune checkpoint inhibitor efficacy. In conclusion, we employed a refined ROI sampling protocol involving three representative solid tumor regions, effectively minimizing heterogeneity effects while maintaining clinical practicality. While the methodology of localized ROI analysis on DWI has been previously documented in other oncological contexts, such as differentiating glioblastoma multiforme from metastatic brain tumors (28), its application and validation as a predictive biomarker specifically for PD-1 inhibitor response in advanced cervical cancer provides distinct clinical utility.

The biological rationale underlying the association between low sADC and poor outcomes in immunotherapy remains to be fully elucidated. Low sADC values reflect high tumor cellularity and restricted water diffusion, which may be indicative of a densely packed tumor microenvironment. This physical barrier, often accompanied by a high stromal content and abnormal vasculature, could impede the infiltration and intratumoral distribution of cytotoxic T lymphocytes (CTLs), a prerequisite for effective immune checkpoint blockade (29, 30). Furthermore, highly cellular tumors may harbor a more immunosuppressive microenvironment, characterized by an abundance of regulatory T cells (Tregs), myeloid-derived suppressor cells (MDSCs), and M2 macrophages, which collectively dampen anti-tumor immune responses (31). Thus, the sADC value may serve as an imaging surrogate for these unfavorable immunological features, explaining its predictive power for immunotherapy resistance.

Compared to invasive biomarkers such as PD-L1 expression and TMB, which require tumor tissue sampling and are subject to spatial heterogeneity and sampling bias, sADC offers a significant advantage as a completely non-invasive, cost-effective, and readily available imaging biomarker that can be assessed at baseline and potentially monitored longitudinally. While sADC reflects the physical and cellular architecture of the tumor, it may provide complementary information to molecular biomarkers like PD-L1. Future studies integrating sADC with genomic and immunological markers could pave the way for multimodal predictive models, enabling more precise patient selection for personalized immunotherapy.

However, this study has several limitations. Firstly, the modest sample size may introduce selection bias. Furthermore, the single-center, retrospective design inherently carries risks of selection bias and unmeasured confounding factors, which may limit the generalizability of our findings. Future multicenter validation studies with larger cohorts are warranted to enhance the reliability and generalizability of these findings. Secondly, the analysis was restricted to pretreatment DWI data without assessing longitudinal ADC changes during therapy. Dynamic monitoring of ADC values may provide more comprehensive information for evaluating the efficacy of immunotherapy. Furthermore, future investigations should explore the integration of multimodal biomarkers, such as combining sADC with the PD-L1 combined positive score (CPS), TMB, and microsatellite instability (MSI), to develop precision prognostic models. The integration of imaging biomarkers and micro-environmental molecular profiles could ultimately advance personalized immunotherapy paradigms for cervical cancer.

## Conclusions

This retrospective study demonstrates that the substantial tumor ADC (excluding necrotic, cystic, and vascular regions) was associated with prognosis in cervical cancer patients receiving PD-1 inhibitor therapy, suggesting sADC as a promising, non-invasive imaging biomarker to stratify patients for personalized immunotherapy.

## Data Availability

The datasets generated and/or analyzed during the current study are not publicly available due to patient privacy and ethical restrictions but are available from the corresponding author on reasonable request.
