# Supplementary material for: Tumor apparent diffusion coefficient as a predictive marker for PD-1 inhibitor outcome in advanced cervical cancer: a retrospective study
Source: Front Oncol. 2026 Mar 27;16:1769071. doi: 10.3389/fonc.2026.1769071 (PMC13065691; doi:10.3389/fonc.2026.1769071)
Supplement: Supplementary file 1 [file DataSheet1.docx]

**Supplementary Materials**

**Supplementary Tables**

**Table S1** Tumor responses in the high-sADC and low-sADC groups.

| Tumor response | High-sADC(n = 91) | Low-sADC(n = 76) | *P* value |
| --- | --- | --- | --- |
| Responders | 23 (25.3%) | 29 (38.2%) |  |
| Non-responders | 68 (74.7%) | 47 (61.8%) |  |
| ORR (%) | 25.3% | 38.2% | 0.073 |
| DCR (%) | 87.9% | 81.6% | 0.253 |

Note: sADC, substantial tumor apparent diffusion coefficient; ORR, objective response rate; DCR, disease control rate.

**Table S2** Univariate and multivariate Cox proportional hazards analyses for PFS in cervical cancer patients.

| Variables | Univariate analysis | |  | Multivariate analysis | |
| --- | --- | --- | --- | --- | --- |
|  | Hazard ratio (95% CI) | *P* value |  | Hazard ratio (95% CI) | *P* value |
| Age |  |  |  |  |  |
| < 50 years | Reference |  |  |  |  |
| ≥ 50 years | 1.04 (0.64 - 1.69) | 0.888 |  |  |  |
| BMI |  |  |  |  |  |
| < 25 kg/m^2^ | Reference |  |  |  |  |
| ≥ 25 kg/m^2^ | 0.89 (0.53- 1.50) | 0.668 |  |  |  |
| FIGO stage |  |  |  |  |  |
| II | Reference |  |  | Reference |  |
| III | 3.42 (1.65 - 7.12) | **< 0.001** |  | 3.63 (1.73 - 7.63) | **< 0.001** |
| IV | 3.76 (1.80 - 7.82) | **< 0.001** |  | 3.98 (1.90 - 8.34) | **< 0.001** |
| Pathological type |  |  |  |  |  |
| Squamous cell carcinoma | Reference |  |  | Reference |  |
| Adenocarcinoma | 1.17 (0.68 - 2.01) | 0.581 |  | 1.31 (0.75 - 2.29) | 0.339 |
| Others | 2.38 (1.17 - 4.86) | **0.017** |  | 2.38 (1.12 - 5.07) | **0.024** |
| Diabetes |  |  |  |  |  |
| No | Reference |  |  |  |  |
| Yes | 1.77 (0.77 - 4.06) | 0.181 |  |  |  |
| Hypertension |  |  |  |  |  |
| No | Reference |  |  |  |  |
| Yes | 0.77 (0.38 - 1.53) | 0.454 |  |  |  |
| TG | 0.90 (0.73 - 1.10) | 0.306 |  |  |  |
| TB | 0.97 (0.92 - 1.02) | 0.221 |  |  |  |
| Albumin | 0.97 (0.92 - 1.03) | 0.288 |  |  |  |
| NLR |  |  |  |  |  |
| < 4.1 | Reference |  |  | Reference |  |
| ≥ 4.1 | 1.80 (1.16 - 2.78) | **0.009** |  | 1.17 (0.67 - 2.05) | 0.586 |
| PLR |  |  |  |  |  |
| < 271.1 | Reference |  |  |  |  |
| ≥ 271.1 | 1.31 (0.82 - 2.10) | 0.258 |  |  |  |
| MLR |  |  |  |  |  |
| < 0.6 | Reference |  |  |  |  |
| ≥ 0.6 | 1.16 (0.64 - 2.10) | 0.621 |  |  |  |
| SII |  |  |  |  |  |
| < 820.8 | Reference |  |  | Reference |  |
| ≥ 820.8 | 2.33 (1.50 - 3.60) | **< 0.001** |  | 1.89 (1.08 - 3.30) | **0.027** |
| Treatment pattern |  |  |  |  |  |
| PD-1 + chemotherapy | Reference |  |  |  |  |
| PD-1 + radiochemotherapy | 0.625 (0.338 – 1.156) | 0.134 |  |  |  |
| PD-1 + chemotherapy +  anti-angiogenic therapy | 0.798 (0.408 – 1.564) | 0.512 |  |  |  |
| sADC Group |  |  |  |  |  |
| High-sADC | Reference |  |  | Reference |  |
| Low-sADC | 1.83 (1.19 - 2.81) | **0.006** |  | 1.70 (1.08 - 2.67) | **0.021** |

Note: PFS, progression-free survival; HR, hazard ratio; CI, confidence interval; BMI, body mass index; FIGO, International Federation of Gynecology and Obstetrics; TG, triglyceride; TB, total bilirubin; NLR, neutrophil to lymphocyte ratio; PLR, platelet to lymphocyte ratio; MLR, monocyte to lymphocyte ratio; SII, systemic immune-inflammation index; PD-1, programmed death-1; sADC, substantial tumor apparent diffusion coefficient.

**Supplementary Figures**

**Figure S1**


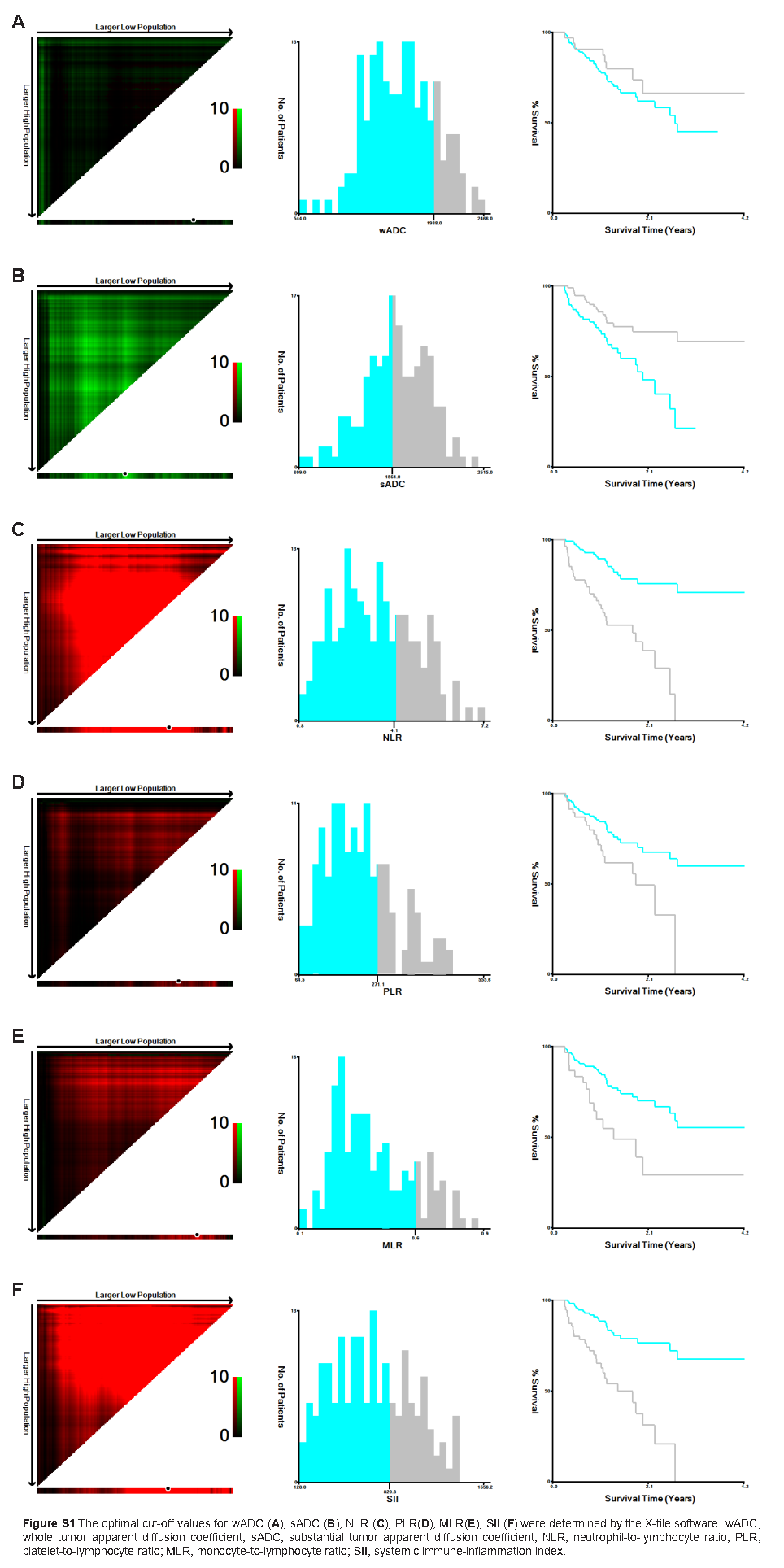


**Figure S2**

**
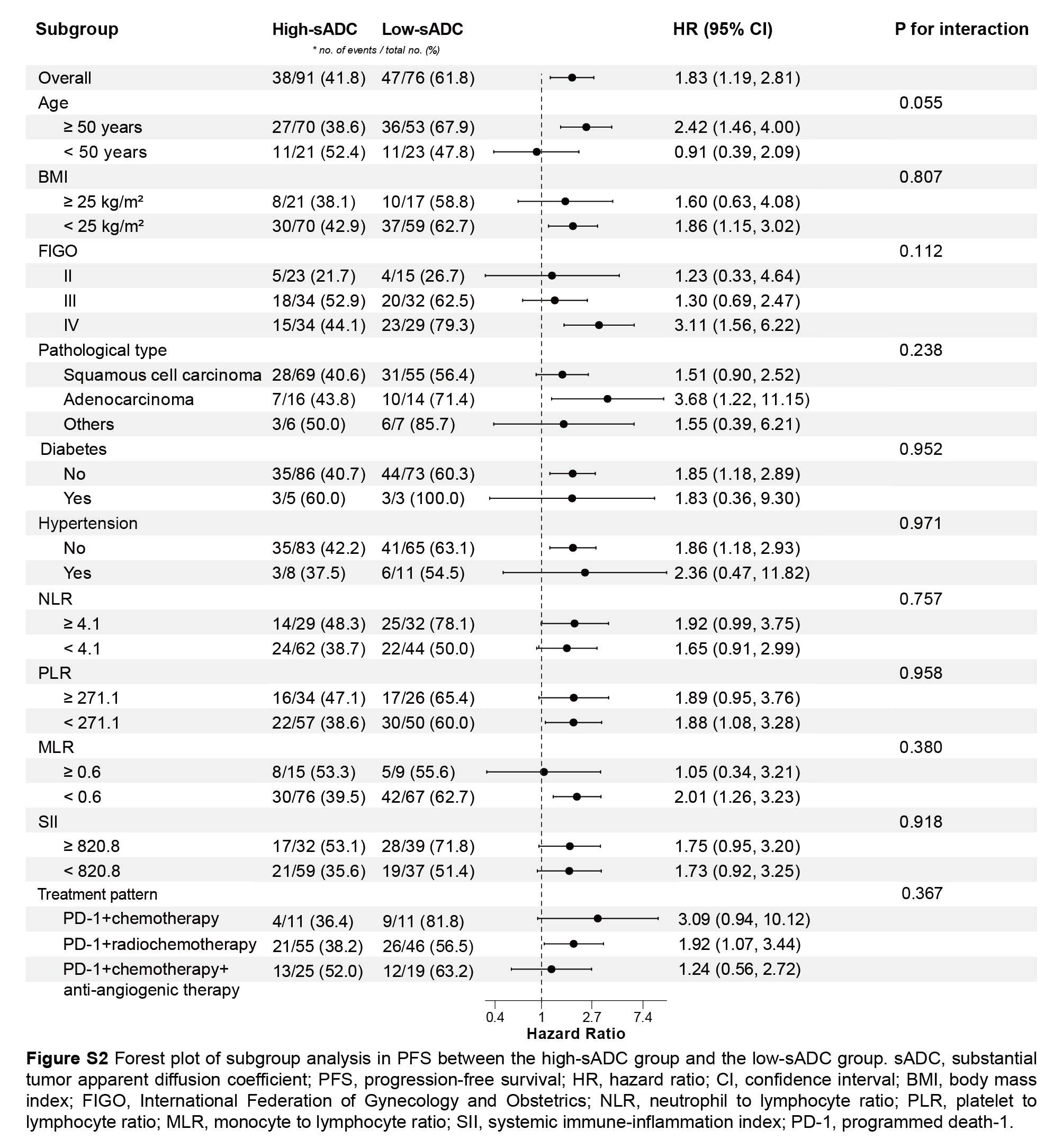
**
